# An Exploratory Study on a New Method for Nutritional Status Assessment in Patients with Chronic Kidney Disease

**DOI:** 10.3390/nu15112640

**Published:** 2023-06-05

**Authors:** Yayong Luo, Hui Huang, Qian Wang, Wenwen Lin, Shuwei Duan, Jianhui Zhou, Jing Huang, Weiguang Zhang, Ying Zheng, Li Tang, Xueying Cao, Jian Yang, Li Zhang, Yong Wang, Jie Wu, Guangyan Cai, Zheyi Dong, Xiangmei Chen

**Affiliations:** 1 Department of Nephrology, First Medical Center of Chinese PLA General Hospital, Nephrology Institute of the Chinese People’s Liberation Army, National Key Laboratory of Kidney Diseases, National Clinical Research Center for Kidney Diseases, Beijing Key Laboratory of Kidney Disease Research, Beijing 100853, China; 2 School of Clinical Medicine, Guangdong Pharmaceutical University, Guangzhou 510006, China

**Keywords:** chronic kidney disease, malnutrition, nutritional assessment methods, subjective global assessment

## Abstract

Malnutrition is a risk factor for disease progression and poor prognosis in chronic kidney disease (CKD). However, the complexity of nutritional status assessment limits its clinical application. This study explored a new method of nutritional assessment in CKD (stage 1–5) patients using the Subjective Global Assessment (SGA) as the gold standard and evaluated its applicability. The kappa test was used to analyze the consistency of the Renal Inpatient Nutrition Screening Tool (Renal iNUT) with SGA and protein-energy wasting. Logistic regression analysis was used to analyze the risk factors of CKD malnutrition and calculate the prediction probability of multiple indicators combined for the diagnosis of CKD malnutrition. The receiver operating characteristic curve of the prediction probability was drawn to evaluate its diagnostic efficiency. A total of 161 CKD patients were included in this study. The prevalence of malnutrition according to SGA was 19.9%. The results showed that Renal iNUT had a moderate consistency with SGA and a general consistency with protein-energy wasting. Age > 60 years (odds ratio, OR = 6.78), neutrophil–lymphocyte ratio > 2.62 (OR = 3.862), transferrin < 200 mg/dL (OR = 4.222), phase angle < 4.5° (OR = 7.478), and body fat percentage < 10% (OR = 19.119) were risk factors for malnutrition in patients with CKD. The area under the receiver operating characteristic curve of multiple indicators for the diagnosis of CKD malnutrition was 0.89 (95% confidence interval: 0.834–0.946, *p* < 0.001). This study demonstrated that Renal iNUT has good specificity as a new tool for the nutrition screening of CKD patients, but its sensitivity needs to be optimized. Advanced age, high neutrophil–lymphocyte ratio, low transferrin level, low phase angle, and low body fat percentage are risk factors for malnutrition in patients with CKD. The combination of the above indicators has high diagnostic efficiency in the diagnosis of CKD malnutrition, which may be an objective, simple, and reliable method to evaluate the nutritional status of patients with CKD.

## 1. Introduction

Chronic kidney disease (CKD) is a non-infectious disease with high incidence and poor prognosis. According to the analysis report of the Global Burden of Disease Chronic Kidney Disease Collaboration [1], there were nearly 700 million CKD patients in the world in 2017, and the global prevalence of CKD was about 9.1%. Malnutrition is related to the progression of CKD and is one of the factors for the poor prognosis of patients [2,3]. Studies have shown that malnutrition reduces the response to drug treatment, prolongs the length of hospital stay, and increases the rate of rehospitalization and medical costs, which in turn leads to decreased quality of life and poor prognosis [4,5].

There is no unified standard for the definition of malnutrition in CKD patients, and the existing and validated nutritional assessment tools are rarely used for the nutritional assessment of CKD patients [6]. In 2008, in order to clarify nutrition-related terms and definitions in patients with kidney disease, the International Society of Renal Nutrition and Metabolism (ISRNM) [7] proposed the concept of protein-energy wasting (PEW), that is, a state of nutritional deficiency caused by insufficient body protein and energy reserves. PEW is more commonly used to reflect the nutritional status of end-stage renal disease (ESRD) patients, it has a high incidence in long-term dialysis patients, and it is associated with adverse clinical outcomes [8]. In 2019, Jackson et al. [9] developed a new tool called the Renal Inpatient Nutrition Screening Tool (Renal iNUT), which was considered to have good sensitivity and specificity compared with the Subjective Global Assessment (SGA) and to be more reliable than the Malnutrition Universal Screening Tool (MUST). The Renal iNUT consists of five closed questions, which can be used for rapid nutritional screening and has good clinical practicability. However, whether Renal iNUT is suitable for the nutrition screening of renal inpatients still needs external verification [6]. No studies have been conducted to validate the applicability of Renal iNUT in CKD patients.

The National Kidney Foundation’s Kidney Disease Outcomes Quality Initiative (KDOQI) nutrition guidelines for CKD in 2020 [10] state that more research should be conducted to examine which composite nutritional indicators are suitable for nutritional screening or assessment in non-dialysis patients with CKD, to standardize the methods for CKD nutrition screening, and to focus on reliable composite nutritional indicators for earlier stages of CKD. SGA is a widely recognized universal nutritional status assessment tool in clinical practice, which can predict morbidity and mortality related to malnutrition. It has been validated in different disease populations, including CKD, and is considered the “gold standard” for nutritional status assessment [11]. Besides nutritional screening and assessment tools, nutritional status assessment methods also include objective indicators such as anthropometric measurements, body composition analysis, functional tests, and clinical laboratory indicators [4]. Many studies have demonstrated the role of anthropometric indicators in the assessment of nutritional status and disease prognosis in CKD, including body mass index (BMI) [12,13], triceps skinfold thickness (TSF) [14], mid-arm-muscle circumference (MAMC) [15,16], body fat percentage (BFP) [17] and phase angle (PhA) [18,19,20] measured by bioelectric impedance (BIA), etc. However, it is necessary to combine multiple indicators to comprehensively evaluate nutritional status.

This study aims to verify the applicability of Renal iNUT in the assessment of nutritional status in CKD (stage 1–5) patients, and to explore the related factors of malnutrition in CKD and the diagnostic efficacy of multiple indicators combined in the diagnosis of CKD malnutrition.

## 2. Materials and Methods

### 2.1. Study Population

A total of 187 patients with CKD who were hospitalized in the Department of Nephrology of the First Medical Center of the Chinese People’s Liberation Army General Hospital from March 2022 to September 2022 were enrolled in this study. Inclusion criteria: (1) patients diagnosed with CKD according to the 2012 KDIGO clinical practice guidelines [21]; (2) age ≥ 18 years old; (3) voluntarily signing of the informed consent and cooperation with data collection. Exclusion criteria: (1) patients who had received renal replacement therapy (such as hemodialysis, peritoneal dialysis, kidney transplantation); (2) patients with acute and severe diseases (acute heart failure, acute cerebrovascular disease, etc.) within the past 6 months; (3) patients with a history of severe infection in the past one month; (4) patients with malignant tumors; (5) pregnant or lactating women; (6) patients with incomplete medical records. After exclusion, 161 patients were finally included. This study was conducted in accordance with the Declaration of Helsinki and approved by the Ethics Committee of the Chinese People’s Liberation Army General Hospital (S2022-259-01). All participants provided written informed consent.

### 2.2. Clinical Data Collection

The general data of subjects such as gender, age, height, weight, duration of nephropathy, and history of diabetes mellitus were collected. Venous blood samples were collected after fasting on the second day of admission to determine white blood cells (WBCs), neutrophil–lymphocyte ratio (NLR), C reactive protein (CRP), interleukin-6 (IL-6), hemoglobin, total protein, albumin, blood urea nitrogen (BUN), serum creatinine, estimated glomerular filtration rate (eGFR, calculated using the CKD-EPI formula [22]), cystatin C, uric acid, total cholesterol, triglyceride, fasting blood glucose (FBG), serum calcium, serum potassium, serum phosphorus, high-density lipoprotein cholesterol (HDL-C), low-density lipoprotein cholesterol (LDL-C), prealbumin, transferrin, haptoglobin, etc.

### 2.3. Anthropometry and Body Composition Indicators

The data on BMI, handgrip strength (HGS), calf circumference (CC), TSF, and body composition of the participants were collected, and the specific calculation and measurement methods were as follows:

1. BMI was calculated by dividing the weight (in kilograms) by the square of the height (in meters).
2. HGS: According to the size of the subject’s hand, the handle of the handgrip dynamometer was adjusted, the subject was informed to take the standing position with the arm naturally sagging, and the handgrip dynamometer was grasped with the unilateral hand as hard as possible. The measurement was accurate to 0.1 kg and repeated three times with an interval of 1 min, and the highest value was taken as the HGS value.
3. CC: The maximum circumference was measured with a tape measure at the right calf of the subject in a sitting position with feet on the floor and knees bent at 90°. The measurement was repeated twice and averaged with an accuracy of 0.1 cm.
4. TSF: The subject’s arm was naturally pendulous, the surveyor pinched the sebum at the midpoint of the dorsal upper arm of the subject with the left thumb and index finger, and they then measured the skinfold thickness with a sebum skinfold caliper at a distance of 1 cm from the finger pinching site. The tip of the caliper was made to fully clamp the skinfold, the results after the pointer came to a complete stop were read and recorded immediately, and the measurements were repeated three times and averaged with an accuracy of 0.1 mm.
5. The body composition indicators of the subjects were collected by the InbodyS10 body composition analyzer, including mid-arm circumference (MAC), MAMC, skeletal muscle mass index (SMI), body protein, body inorganic salts, body bone mineral content, body fat, BFP, and PhA. Before measurement, it was confirmed whether there was no pacemaker or metal implant in the subject, and the subject was informed to take the supine position, exposing the bilateral fingers and ankles. The relevant information of the subject was input on the instrument operation panel, the upper-limb electrode clips were clamped on the thumb and middle finger of the subject, the lower-limb electrode clips were clamped on the ankle of the subject, and the measurement was started after confirming that the electrode clips were properly clamped. After completion of the measurement, the electrode clips were removed, and the body composition report was read.

### 2.4. Nutritional Status Assessment

The program content for SGA, PEW, and Renal iNUT was gathered by trained clinicians. The assessment of SGA included five items of medical history (weight change, dietary intake change, gastrointestinal symptoms, functional capacity, and metabolic demand) and three items of physical examination (subcutaneous fat loss, muscle wasting, and edema) [23]. Participants rated as grade A were classified as “non-malnutrition” and those rated as grade B or C were classified as “malnutrition”.

According to the diagnostic criteria of PEW formulated by ISRNM [7], it includes four categories: serum chemistry, body mass, muscle mass, and dietary intake. PEW can be diagnosed if three categories are met (at least one item of each category satisfies the criteria). The data on dietary protein and energy intake were collected by the 24 h dietary recall and then were calculated according to the Dietary Reference Intakes for Chinese [24].

Renal iNUT [9] includes five issues: unintentional weight loss, BMI, nutritional supplements, and changes in food intake and appetite. The nutritional status of the subjects was evaluated based on the total score result, and each issue could be rated as a “0 score ” or “1 score ”; subjects with a score ≤1 were considered as “non-malnutrition” and those with a score ≥2 were considered as “malnutrition”.

### 2.5. Statistical Analysis

The measurement data with the normal distribution were described by mean ± standard deviation and were analyzed using Student’s *t*-tests. Non-normally distributed data were expressed as the median with interquartile range and analyzed using the Mann–Whitney U test. Count variables were expressed as frequency and percentages and analyzed using chi-square or Fisher’s exact test. The kappa test was used to analyze the consistency of Renal iNUT with SGA and PEW (kappa value: 0–0.2 for poor consistency, 0.21–0.4 for general consistency, 0.41–0.6 for moderate consistency, 0.61–0.8 for good consistency, >0.8 for almost complete consistency). Logistic regression analysis was used to analyze the related factors of CKD malnutrition and calculate the prediction probability of multiple indicators combined for the diagnosis of CKD malnutrition. The receiver operating characteristic (ROC) curve of the prediction probability was drawn to evaluate its diagnostic efficiency. The statistical analyses were performed using SPSS version 26.0 for Mac (SPSS, Chicago, IL, USA). Statistical significance was set at *p* < 0.05.

## 3. Results

### 3.1. Subject Screening and CKD Staging

**Figure 1:**
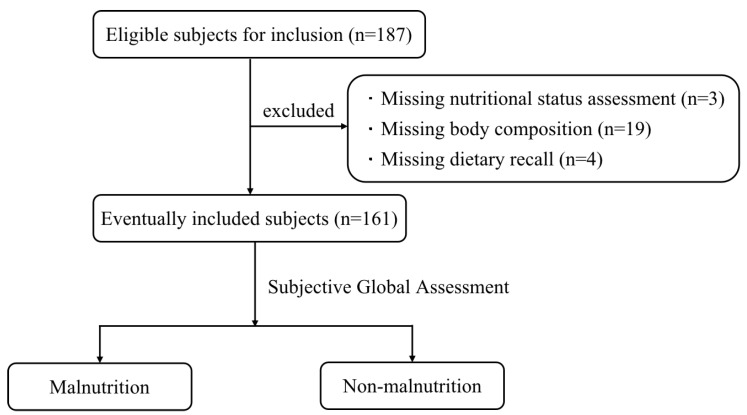
Flow chart of subject screening.

A total of 187 CKD patients hospitalized in the Department of Nephrology of the First Medical Center of the Chinese People’s Liberation Army General Hospital from March 2022 to September 2022 were selected. After excluding 3 cases without nutritional status assessment, 19 cases without body composition, and 4 cases missing dietary recall, 161 subjects were finally included. The subject screening process is shown in Figure 1.

**Figure 2:**
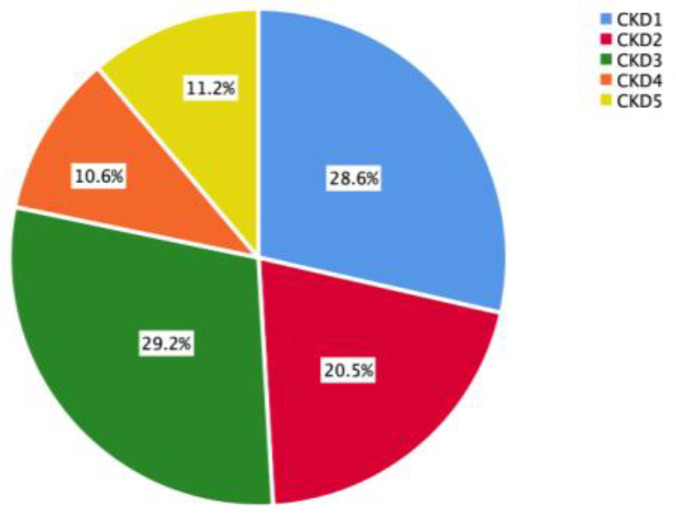
CKD stage of the study population.

Among the 161 CKD patients, 46 (28.6%) were at the CKD1 stage, 33 (20.5%) at the CKD2 stage, 47 (29.2%) at the CKD3 stage, 17 (10.6%) at the CKD4 stage, and 18 (11.2%) at the CKD5 stage. As shown in Figure 2, patients with stage CKD1-3 accounted for a large proportion of the study population.

### 3.2. Assessment of Nutritional Status of the Study Population

The prevalence of malnutrition diagnosed according to SGA, PEW, and Renal iNUT was 19.9% (*n* = 32), 19.9% (*n* = 32), and 21.7% (*n* = 35), respectively, and there was no significant difference between the gender groups (*p* > 0.05), as shown in Table 1.

According to SGA, all subjects (100%) had a low stress of metabolic demand, 41.6% (*n* = 67) had reduced functional capacity, 37.9% (*n* = 61) had a loss of subcutaneous fat, and 23.6% (*n* = 38) had edema. There were 29 (18%), 20 (12.4%), and 11 (6.8%) cases of muscle wasting, reduced dietary intake, and weight loss, respectively. None of the SGA items showed statistical differences between gender group comparisons.

In the diagnosis of PEW, the majority (*n* = 116, 72%) of the participants had inadequate dietary intake, and 59% (*n* = 95) had subnormal serum chemistry. Fifty-five participants (34.2%) had decreased body mass and 17 (10.6%) had muscle mass loss. The proportion of participants with decreased body mass was higher in females than in males (46.8% vs. 26.3%, *p* = 0.008).

In the assessment of Renal iNUT, 52.8% (*n* = 85) of the subjects took nutritional supplements, and 21 (13%), 18 (11.2%), and 16 (9.9%) subjects had unintentional weight loss, poor appetite, and a BMI less than or equal to 20 kg/m^2^, respectively. Among gender groups, the proportion of female subjects with a BMI less than or equal to 20 kg/m^2^ was significantly higher than that of male subjects (21% vs. 3%, *p* < 0.001).

### 3.3. Consistency Test between Renal iNUT and SGA and PEW

The results of the kappa test showed that Renal iNUT had moderate consistency with SGA (Kappa = 0.454, *p* < 0.001). Compared with SGA, Renal iNUT had a sensitivity of 59.4%, a specificity of 87.6%, a positive predictive value of 54.3%, and a negative predictive value of 89.7% in diagnosing malnutrition (Table 2), while the consistency between Renal iNUT and PEW was general (Kappa = 0.303, *p* < 0.001). Compared with PEW, Renal iNUT had a sensitivity of 46.9%, a specificity of 84.5%, a positive predictive value of 42.9%, and a negative predictive value of 86.5% for diagnosing malnutrition (Table 3).

### 3.4. Comparison of Clinical Characteristics and Anthropometric Parameters of Malnutrition and Non-Malnutrition Subjects

According to the SGA, the subjects were classified as malnutrition and non-malnutrition. Compared with the non-malnutrition group, the levels of NLR, IL-6, BUN, cystatin C, and serum phosphorus in the malnutrition group were higher, while the levels of hemoglobin, total protein, albumin, prealbumin, transferrin, eGFR, serum calcium, and dietary energy intake were lower (*p* < 0.05), as shown in Table 4. There were no significant differences in WBC, CRP, haptoglobin, serum creatinine, uric acid, blood lipids, FBG, serum potassium, and dietary protein intake between the two groups (*p* > 0.05).

The BMI, HGS, CC, MAC, MAMC, body fat, and PhA were lower in the malnutrition group than in the non-malnutrition group (*p* < 0.05). There were no statistically significant differences in TSF, SMI, body protein, body inorganic salts, body bone mineral content, and BFP between the two groups (Table 5).

### 3.5. Logistic Regression Analysis of Malnutrition in CKD

The indicators with *p* < 0.1 in univariate analysis were used for multivariate logistic regression analysis with SGA assessment results as the dependent variable. Combined with the results of collinearity diagnosis, age, NLR, IL-6, hemoglobin, albumin, prealbumin, transferrin, BUN, cystatin C, blood calcium, blood phosphorus, BMI, CC, HGS, PhA, and BFP were finally included in the analysis, and gender was included to control the confounding factors (Appendix A Table A2). According to the clinical examination and measurement standards, the above continuous variables were transformed into categorical variables and assigned values (Appendix A Table A3). The multivariate logistic regression analysis was conducted by using the forward stepwise method, and the results showed that age > 60 years (odds ratio, OR = 6.78), NLR > 2.62 (OR = 3.862), transferrin < 200 mg/dL (OR = 4.222), PhA < 4.5° (OR = 7.478), and BFP < 10% (OR = 19.119) were risk factors for malnutrition in CKD patients (Table 6).

### 3.6. ROC Curve of Multiple Indicators Combined for the Diagnosis of CKD Malnutrition

**Figure 3:**
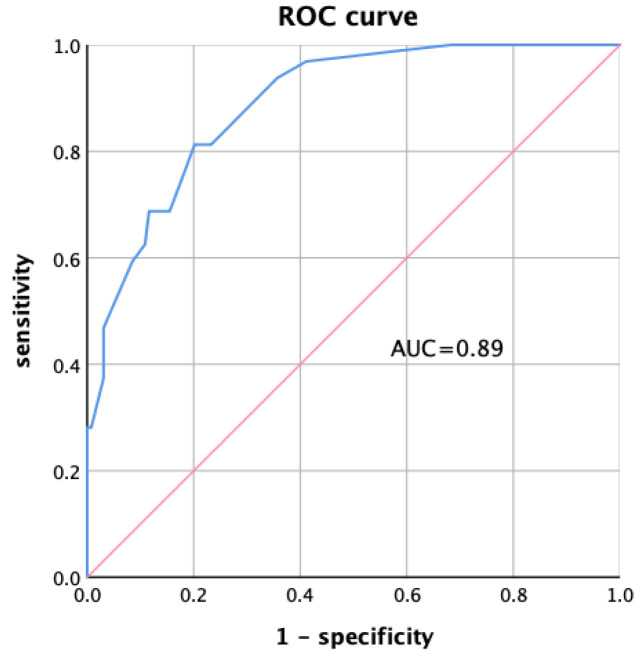
ROC curve of multiple indicators combined diagnosis.

According to the results of multivariate logistic regression analysis of CKD malnutrition, the score Logit(*P*) and the prediction probability *P* of multiple indicators combined for the diagnosis of CKD malnutrition were calculated (Equations (1) and (2)). The area under the curve (AUC) of the prediction probability was 0.89 (95% confidence interval (CI): 0.834–0.946, *p* < 0.001), as shown in Figure 3.

Logit (*P*) = −4.675 + 1.914 × age (≤60 years = 0, >60 years = 1)           + 1.351 × neutrophil-lymphocyte ratio (≤2.62 = 0, >2.62 = 1)         + 1.44 × transferrin (≥200 mg/dL = 0, <200 mg/dL = 1)    + 2.012 × phase Angle (≥4.5° = 0, <4.5° = 1)       + 2.951 × body fat percentage (≥10% = 0, <10% = 1)(1)

*P* = e^Logit(*P*)^/(1 + e^Logit(*P*)^) (2)

## 4. Discussion

Malnutrition is usually defined as “a state of decreased physical and mental function and impaired clinical outcomes due to changes in body composition (fat free mass) and body cell mass caused by lack of nutrient intake”, but there is no clear and generally accepted diagnostic criteria for malnutrition [25]. In 2017, the European Society for Clinical Nutrition and Metabolism (ESPEN) [12] defined “clinical nutrition” and proposed that clinical nutrition is a discipline of prevention, diagnosis, and management of nutritional and metabolic changes related to acute and chronic diseases and disorders caused by a deficiency or excess of energy and nutrients, and classified it as malnutrition, overnutrition (overweight or obesity), micronutrient deficiency or excess, sarcopenia, frailty, and refeeding syndrome. Malnutrition can be further divided into disease-related malnutrition (with or without inflammation) and non-disease-related malnutrition due to inadequate diet, socioeconomic, or psychological factors [12].

It has been reported that the prevalence of malnutrition in patients with stage CKD3-5 ranges from 11% to 54% worldwide [26], and the prevalence increased with the progression of the CKD stage [27]. In China, the prevalence of CKD malnutrition is 22.5–58.5% [28]. In this study, patients with CKD stage 1–3 accounted for a large proportion (78.3%), and the prevalence of malnutrition in CKD stage 1–5 was 13–33.3% (Appendix A Table A1), which is basically consistent with literature reports. The occurrence of malnutrition in CKD is multifactorial and complex, which may be the reason for the high prevalence of CKD malnutrition. Different CKD stages have different dietary intake requirements. A low-protein diet (0.55–0.60 g/kg/d) can reduce the risk of ESRD and death in patients with CKD and, combined with keto-acid analogs, may reduce the incidence of malnutrition [10]. In addition to insufficient nutrient intake, CKD malnutrition is also related to chronic inflammation, intestinal flora imbalance, metabolic acidosis, insulin resistance, infection, and oxidative stress [6].

SGA is considered to be the most effective tool for nutrition assessment of hospitalized patients, and it was commonly used as the “gold standard” in the study [29]. PEW is mainly used to describe a state of nutritional deficiency in patients with kidney disease, in which protein and energy reserves are reduced due to insufficient intake or increased demand or nutrient loss, which cannot meet the metabolic needs of the body [28]. Renal iNUT is a nutrition screening tool developed by Jackson et al. for hospitalized patients with kidney disease, which is more effective and reliable than MUST [9]. In this study, we compared the Renal iNUT with SGA and PEW to verify its applicability in the nutrition screening of CKD-hospitalized patients. The results of the Kappa test showed that the consistency between Renal iNUT and SGA was moderate (Kappa = 0.454), while the consistency between Renal iNUT and PEW was general (Kappa = 0.303). Compared with SGA, Renal iNUT had a better specificity (87.6%) and negative predictive value (89.7%), but a lower sensitivity (59.4%) and positive predictive value (54.3%). Renal iNUT also showed a high specificity (84.5%) and negative predictive value (86.5%) when compared with PEW. Renal iNUT can effectively screen patients without malnutrition, but the accuracy in screening malnutrition patients is insufficient, which may be related to the fact that the content of Renal iNUT is too simple and has an unclear definition. For example, the Renal iNUT assessment does not include a specific value or proportion of weight loss in the “unintentional weight loss” category. This study found that the proportion of female patients was significantly higher than that of males in the “BMI ≤ 20 kg/m^2^” project (21% vs. 3%). It is worth exploring whether gender differences should be considered in the definition of BMI in the nutrition assessment.

In the multivariate logistic regression analysis, age, NLR, transferrin, PhA, and BFP were associated with CKD malnutrition, and the combination of the above indicators had high diagnostic efficacy in the diagnosis of CKD malnutrition (AUC = 0.89, *p* < 0.001). The diagnosis of multiple indicators combined may be a simple, objective, and reliable method to evaluate the nutritional status of CKD.

Aging is one of the risk factors for malnutrition. The prevalence of malnutrition in elderly inpatients is significantly higher than that in young patients. It was reported that the prevalence of malnutrition was 1.2–2.3 times higher in patients over 65 years old than in those under 65 years old [30]. Various factors related to aging may lead to the occurrence of malnutrition, such as decreased appetite, delayed gastric emptying, decreased taste and smell, and changes in hormone levels [31]. A multicenter prospective cohort study found that the risk of malnutrition in CKD increased with age [32]. In this study, patients with malnutrition were significantly older than those without malnutrition (*p* < 0.001). The risk of malnutrition in CKD patients over 60 years old was 6.78 times higher than that in those under 60 years old (95%CI: 2.252–20.413, *p* = 0.001). The study by Xi et al. [33] also found that age was an independent risk factor for malnutrition in CKD patients.

NLR is a biomarker reflecting the balance between two aspects of the immune system: acute and chronic inflammation (such as neutrophil count) and adaptive immunity (lymphocyte count) [34]. NLR has proven to be an independent prognostic factor for morbidity and mortality in many diseases, and elevated NLR was associated with mortality in patients with heart disease, chronic lower-respiratory-tract disease, influenza or pneumonia, and kidney disease, but its normal cut-off value remains controversial [34,35]. Han et al. [36] found that high NLR was an independent risk factor for malnutrition in CKD (OR = 1.393, *p* = 0.011), and NLR ≥ 2.62 could be used to identify CKD malnutrition. This study similarly found that NLR was higher in malnutrition CKD patients than in non-malnutrition patients. Taking 2.62 as the cut-off value of NLR, the risk of malnutrition in CKD patients with NLR > 2.62 was 3.862 times higher than that in patients with NLR ≤ 2.62 (95%CI: 1.344–11.104, *p* = 0.012).

Both hemoglobin and transferrin are indicators of anemia. The study by Ucha et al. [37] showed that the malnutrition–inflammation complex syndrome in CKD patients was associated with anemia. Aggarwal et al. [38] found that hemoglobin level was negatively correlated with CKD malnutrition, and total iron binding capacity was an independent related factor for CKD malnutrition, which is consistent with the results of our study. In this study, the hemoglobin level of malnutrition was significantly lower than that of non-malnutrition (104.75 g/L vs. 121.04 g/L, *p* = 0.001), there was also a significant difference between the two groups in transferrin (167 mg/dL vs. 190.08 mg/dL, *p* < 0.001), and low transferrin level (<200 mg/dL) was a risk factor for malnutrition in CKD.

Anthropometric measurements are integral parts of the CKD nutritional assessment. BMI is commonly used to assess nutritional status and obesity. In the 2015 consensus of the European Society of Clinical Nutrition and Metabolism [25], a BMI of less than 18.5 kg/m^2^ was used as a diagnostic criterion for malnutrition, and the BMI cutoffs for assessing malnutrition were divided according to age (<70 years, 20 kg/m^2^; ≥70 years old, 22 kg/m^2^) but need to consider racial and regional differences. TSF could be used to reflect the reserve of human subcutaneous fat, and MAC and MAMC are indicators of muscle consumption. MAMC and computed tomography have good consistency in assessing muscle mass in CKD patients [39]. Cuppari et al. [40] found that BMI, TSF, MAC, and MAMC were lower in CKD patients with poorer SGA scores, and there was a moderate to good agreement between the anthropometric parameters and the presence of PEW assessed by SGA. Our study similarly revealed that BMI, MAC, and MAMC in the malnutrition group were lower than those with non-malnutrition. Many studies have shown that indicators measured by BIA can be used to reflect the nutritional status of CKD and provide information on disease progression and clinical prognosis, such as body water, BFP, and PhA [17,18,19,41]. Wang et al. [19] showed that the PhA was negatively correlated with the malnutrition inflammation score (r = −0.475, *p* < 0.001). Our study also found that PhA was significantly lower in the malnutrition group than in the non-malnutrition group (4.88 vs. 6.03, *p* < 0.001). Studies by Bansal et al. [42] and Barril et al. [43] demonstrated that lower phase angles were associated with increased mortality risk in CKD patients. Shen et al. [17] showed that BFP is related to BMI, CC, nutritional markers, and CRP, and can predict all-cause mortality in patients with advanced CKD. In this study, lower levels of PhA (<4.5°) and BFP (<10%) were risk factors for malnutrition in CKD patients.

In order to provide a relatively objective, simple, and reliable method for the assessment of nutrition status in CKD patients, this study verified the applicability of renal iNUT in the CKD population for the first time. Moreover, we collected comprehensive data including clinical laboratory indicators, anthropometry, and body composition indicators to describe the nutritional status of CKD patients and evaluate the diagnostic value of multiple indicators combined in the diagnosis of CKD malnutrition. There are some limitations in this study. First, this study is a single-center study, and the study population is relatively limited. Multi-center studies can be carried out to verify the method of multiple indicators combined for the diagnosis of CKD malnutrition in a wider population, to make the results of the study more representative in the future. Second, it is necessary to expand the sample size and reduce sample bias to make the study results more reliable. At the same time, a follow-up cohort can be established to further explore the relationship between the related factors of CKD malnutrition and the progression and prognosis of CKD.

## 5. Conclusions

This study shows that Renal iNUT has good specificity as a new tool for nutrition screening in CKD patients, but its sensitivity needs to be optimized. Advanced age (>60 years), high NLR (≥2.62), low transferrin level (<200 mg/dL), low PhA (<4.5°), and low BFP (<10%) are risk factors for CKD malnutrition. The combination of the above indicators has high diagnostic efficiency in the diagnosis of CKD malnutrition. The diagnosis of multiple indicators combined may be an objective, simple, and reliable method to evaluate the nutritional status of CKD patients.

## Figures and Tables

**Table 1:** Nutritional status assessment of the study population.

Assessment Tool	Item	Total (*n*, %)	Male (*n*, %)	Female (*n*, %)	*p* Value
SGA	Weight change	11 (6.8%)	6 (6.1%)	5 (8.1%)	0.75
	Dietary intake change	20 (12.4%)	15 (15.2%)	5 (8.1%)	0.185
	Gastrointestinal symptoms	13 (8.1%)	6 (6.1%)	7 (11.3%)	0.236
	Functional capacity	67 (41.6%)	38 (38.4%)	29 (46.8%)	0.293
	Metabolic demand	161 (100%)	99 (100%)	62 (100%)	–
	Subcutaneous fat loss	61 (37.9%)	36 (36.4%)	25 (40.3%)	0.614
	Muscle wasting	29 (18%)	19 (19.2%)	10 (16.1%)	0.623
	Edema	38 (23.6%)	22 (22.2%)	16 (25.8%)	0.602
	Result of SGA	32 (19.9%)	18 (18.2%)	14 (22.6%)	0.496
PEW	Serum chemistry	95 (59%)	54 (54.5%)	41 (66.1%)	0.146
	Body mass	55 (34.2%)	26 (26.3%)	29 (46.8%)	0.008
	Muscle mass	17 (10.6%)	10 (10.1%)	7 (11.3%)	0.811
	Dietary intake	116 (72%)	68 (68.7%)	48 (77.4%)	0.23
	Result of PEW	32 (19.9%)	17 (17.2%)	15 (24.2%)	0.277
Renal iNUT	Unintentional weight loss	21 (13%)	15 (15.2%)	6 (9.7%)	0.316
	BMI ≤ 20 kg/m^2^	16 (9.9%)	3 (3%)	13 (21%)	<0.001
	Nutritional supplements	85 (52.8%)	47 (47.5%)	38 (61.3%)	0.087
	Food intake	20 (12.4%)	15 (15.2%)	5 (8.1%)	0.185
	Appetite	18 (11.2%)	12 (12.1%)	6 (9.7%)	0.632
	Result of Renal iNUT	35 (21.7%)	21 (21.2%)	14 (22.6%)	0.838

SGA, Subjective Global Assessment; PEW, protein-energy wasting; Renal iNUT, Renal Inpatient Nutrition Screening Tool; BMI, body mass index.

**Table 2:** Assessment result of Renal iNUT and SGA.

Renal iNUT	SGA		Sum
	Malnutrition	Non-Malnutrition	
Malnutrition	19	16	35
Non-malnutrition	13	113	126
Sum	32	129	161

SGA, Subjective Global Assessment; Renal iNUT, Renal Inpatient Nutrition Screening Tool.

**Table 3:** Assessment result of Renal iNUT and PEW.

Renal iNUT	PEW		Sum
	PEW	Non-PEW	
Malnutrition	15	20	35
Non-malnutrition	17	109	126
Sum	32	129	161

PEW, protein-energy wasting; Renal iNUT, Renal Inpatient Nutrition Screening Tool.

**Table 4:** Comparison of clinical characteristics between malnutrition and non-malnutrition CKD patients.

Variables	Non-Malnutrition (*n* = 129)	Malnutrition (*n* = 32)	*p* Value
Age (years)	47 (37.5, 59)	61.5 (54, 69)	<0.001
Male, *n* (%)	81 (62.8%)	18 (56.3%)	0.496
CKD course (months)	20 (9, 62)	19.5 (9.25, 49.5)	0.821
Diabetes, *n* (%)	57 (44.2%)	18 (56.3%)	0.221
Hypertension, *n* (%)	95 (73.6%)	26 (81.3%)	0.373
WBC (×10^9^/L)	6.79 (5.71, 8.1)	6.03 (4.98, 8.24)	0.199
NLR	2.13 (1.58, 2.86)	2.56 (1.81, 3.32)	0.07
CRP (mg/dL)	0.09 (0.05, 0.16)	0.1 (0.05, 0.21)	0.625
IL-6 (pg/mL)	2.45 (2, 4.02)	3.48 (2.06, 6.11)	0.023
Hemoglobin (g/L)	121.04 ± 24.57	104.75 ± 18.59	0.001
Total protein (g/L)	58.59 ± 9.23	52.69 ± 10.01	0.002
Albumin (g/L)	37.4 (32.5, 41)	31.05 (25.23, 35.95)	<0.001
Prealbumin (mg/dL)	29.18 (24.75, 33.55)	25.6 (20.93, 31.43)	0.034
Transferrin (mg/dL)	190.08 (168.5, 224.5)	167 (143, 186.5)	<0.001
Haptoglobin (mg/dL)	128.03 (84.3, 161)	125 (79.9, 172.5)	0.912
BUN (mmol/L)	7.77 (5.41, 10.15)	9.81 (6.14, 14.61)	0.024
Serum creatinine (umol/L)	109.2 (74.75, 163.25)	122.35 (78.6, 316.58)	0.199
eGFR (mL/min/1.73 m^2^)	61.24 (35.46, 94.43)	42.07 (16.53, 77)	0.043
Cystatin C (mg/L)	1.52 (1.1, 1.9)	1.8 (1.24, 2.91)	0.015
Uric acid (umol/L)	377.07 ± 92.14	346.72 ± 101.06	0.104
Total cholesterol (mmol/L)	4.52 (3.83, 5.4)	4.2 (3.45, 6.51)	0.588
Triglyceride (mmol/L)	1.76 (1.23, 2.4)	1.61 (1.22, 2)	0.391
FBG (mmol/L)	4.64 (4.12, 5.25)	4.8 (4.4, 5.5)	0.164
Serum calcium (mmol/L)	2.18 (2.08, 2.27)	2.09 (1.95, 2.25)	0.017
Serum potassium (mmol/L)	3.89 (3.64, 4.08)	3.85 (3.42, 4.61)	0.997
Serum phosphorus (mmol/L)	1.24 ± 0.21	1.35 ± 0.27	0.012
HDL-C (mmol/L)	1.12 (0.92, 1.3)	1.09 (0.9, 1.51)	0.906
LDL-C (mmol/L)	2.66 (2.09, 3.35)	2.54 (1.92, 4.12)	0.719
Dietary protein (g/kg/d)	0.83 (0.64, 1.15)	0.66 (0.55, 1.06)	0.052
Dietary energy (kcal/kg/d)	19.33 (14.26, 25.85)	16.38 (12.14, 21.04)	0.044

CKD, chronic kidney disease; WBC, white blood cell; NLR, neutrophil–lymphocyte ratio; CRP, C reactive protein; IL-6, interleukin-6; BUN, blood urea nitrogen; eGFR, estimated glomerular filtration rate; FBG, fasting blood glucose; HDL-C, high-density lipoprotein cholesterol; LDL-C, low-density lipoprotein cholesterol.

**Table 5:** Comparison of anthropometric parameters between malnutrition and non-malnutrition CKD patients.

Variables	Non-Malnutrition (*n* = 129)	Malnutrition (*n* = 32)	*p* Value
BMI (kg/m^2^)	25.18 ± 3.71	23.54 ± 3.38	0.024
HGS (kg)	30.24 ± 10.2	25.02 ± 8.08	0.008
CC (cm)	36.56 ± 3.49	35.17 ± 2.76	0.038
TSF (cm)	1.59 ± 0.5	1.46 ± 0.48	0.182
MAC (cm)	29.2 (27.45, 31.3)	27.25 (26.1, 28.95)	0.002
MAMC (cm)	26.1 (24.3, 27.65)	24.05 (23.08, 25.68)	0.002
SMI (kg/m^2^)	8.72 ± 1.4	8.83 ± 1.74	0.74
Body protein (kg)	10.61 ± 2.1	10.11 ± 2.21	0.229
Body inorganic salts (kg)	3.79 ± 0.76	3.61 ± 0.73	0.228
Body bone mineral content (kg)	3.11 (2.7, 3.53)	2.83 (2.5, 3.34)	0.091
Body fat (kg)	16.76 ± 7.12	12.96 ± 8.08	0.009
BFP (%)	23.2 ± 8.04	19.73 ± 12.08	0.131
PhA (°)	6.03 ± 1.03	4.88 ± 1.17	<0.001

BMI, body mass index; HGS, handgrip strength; CC, calf circumference; TSF, triceps skinfold thickness; MAC, mid-arm circumference; MAMC, mid-arm muscle circumference; SMI, skeletal muscle mass index; BFP, body fat percentage; PhA, phase Angle.

**Table 6:** Multivariate logistic regression analysis of malnutrition in CKD.

Variables	B	P	OR	95%CI	
				Lower	Upper
Age	1.914	0.001	6.78	2.252	20.413
NLR	1.351	0.012	3.862	1.344	11.104
Transferrin	1.44	0.036	4.222	1.099	16.218
PhA	2.012	0.001	7.478	2.229	25.093
BFP	2.951	<0.001	19.119	4.404	83.003
Constant	−4.675	<0.001	0.009		

OR, odds ratio; CI, confidence interval; NLR, neutrophil–lymphocyte ratio; PhA, phase Angle; BFP, body fat percentage.

## Data Availability

The data presented in this study are available upon request from the corresponding author.

## Appendix A

**Table A1:** CKD stage and prevalence of malnutrition.

CKD Stage	SGA	
	Malnutrition	Non-Malnutrition
stage 1	6 (13%)	40 (87%)
stage 2	7 (21.2%)	26 (78.8%)
stage 3	8 (17%)	39 (83%)
stage 4	5 (29.4%)	12 (70.6%)
stage 5	6 (33.3%)	12 (66.7%)

CKD, chronic kidney disease; SGA, Subjective Global Assessment.

**Table A2:** Univariate logistic regression analysis of malnutrition in CKD.

Variables	OR	95%CI	*p* Value
Age	1.066	1.03–1.10	<0.001
Gender	0.762	0.348–1.669	0.497
NLR	1.402	1.015–1.938	0.04
IL-6	1.109	0.998–1.233	0.055
Hemoglobin	0.973	0.956–0.99	0.003
Albumin	0.908	0.861–0.958	<0.001
Prealbumin	0.935	0.882–0.992	0.025
Transferrin	0.98	0.97–0.991	<0.001
BUN	1.11	1.029–1.197	0.007
Cystatin C	1.732	1.159–2.587	0.007
Serum calcium	0.028	0.002–0.371	0.007
Serum phosphorus	8.738	1.532–49.826	0.015
BMI	0.879	0.785–0.985	0.026
CC	0.88	0.778–0.995	0.041
HGS	0.94	0.898–0.985	0.009
PhA	0.37	0.242–0.566	<0.001
BFP	0.957	0.915–1.001	0.054

CKD, chronic kidney disease; OR, odds ratio; CI, confidence interval; NLR, neutrophil–lymphocyte ratio; IL-6, interleukin-6; BUN, blood urea nitrogen; BMI, body mass index; CC, calf circumference; HGS, handgrip strength; PhA, phase Angle; BFP, body fat percentage.

**Table A3:** Logistic regression variable assignment.

Variables	Assignment Method
Group	non-malnutrition = 0; malnutrition = 1
Age	≤60 years = 0; >60 years = 1
Gender	female = 0; male = 1
NLR	≤2.62 = 0; >2.62 = 1
IL-6	≤5.9 pg/mL = 0; >5.9 pg/mL = 1
Hemoglobin	male ≤ 137 g/L, female ≤ 116 g/L = 0; male > 137 g/L, female > 116 g/L = 1
Albumin	≥35g/L = 0; <35g/L = 1
Prealbumin	≥20 mg/dL = 0; <20 mg/dL = 1
Transferrin	≥200 mg/dL = 0; <200 mg/dL = 1
BUN	≤7.5 mmol/L = 0; >7.5 mmol/L = 1
Cystatin C	≤1.25 mg/L = 0; >1.25 mg/L = 1
Serum calcium	≥2.09 mmol/L = 0; <2.09 mmol/L = 1
Serum phosphorus	≤1.12 mmol/L = 0; >1.12 mmol/L, ≤1.25 mmol/L = 1; >1.25 mmol/L, ≤1.4 mmol/L = 2; >1.4 mmol/L = 3
BMI	≥20 kg/m^2^ = 0; <20 kg/m^2^ = 1
CC	male ≥ 34 cm, female ≥ 33 cm = 0; male < 34 cm, female < 33 cm = 1
HGS	male ≥ 28 kg, female ≥ 18 kg = 0; male < 28 kg, female < 18 kg = 1
PhA	≥4.5° = 0; <4.5° = 1
BFP	≥10% = 0; <10% = 1

NLR, neutrophil–lymphocyte ratio; IL-6, interleukin-6; BUN, blood urea nitrogen; BMI, body mass index; CC, calf circumference; HGS, handgrip strength; PhA, phase Angle; BFP, body fat percentage.
